# Numerical study on blasting damage of rock with eccentric charge structure

**DOI:** 10.1371/journal.pone.0336825

**Published:** 2026-01-16

**Authors:** Kaihua Liang, Hong Zhao, Yang Li, Weihui Pan, Fuwu Ma, Hai Rong, Nannan Li

**Affiliations:** 1 Shandong Province Research Institute of Coal Geology Planning and Exploration, Jinan, China; 2 Engineering Laboratory of Deep Mine Rockburst Disaster Assessment, Jinan, China; 3 Shandong Taishan Resources Prospecting Group Ltd., Jinan, China; 4 Liaoning Technical University, Fuxin, China; Islamic Azad University Mashhad Branch, IRAN, ISLAMIC REPUBLIC OF

## Abstract

To enhance the safety and efficiency of blasting operations in mines, this study numerically investigates the influence of eccentric versus concentric decoupled charge structures on rock fragmentation under constant charge mass. Using ANSYS/LS-DYNA simulations, the distribution of effective stress (ES) and crack propagation patterns around a single borehole were analyzed. Damage evolution was quantitatively assessed through the damage area ratio (DAR) metric. The results indicate that eccentric charges induce an asymmetric stress field, with ES on the coupled side being up to 2.29 times higher than that on the uncoupled side, and promote vertically symmetrical crack propagation with higher crack density near the charge. The influence range of eccentric charging is limited. Beyond this range, the stress field converges to that produced by concentric charging. Compared to a single charge, multi-charge layouts (double/triple) significantly improve blasting effectiveness, with the optimal structure depending on the number of charges: bottom-eccentric for single or double charges, and concentric for triple charges. These findings provide concrete theoretical guidance for designing controlled blasting schemes in engineering applications such as soft rock blasting, presplitting, and roadway excavation in hard rock formations.

## 1 Introduction

In the realm of roadway construction, the task of excavating rock mass roadways involves handling substantial volumes of material. The borehole blasting method has become the dominant technique for rock excavation, favored for its high efficiency, cost-effectiveness, and adaptability to a wide range of complex scenarios [1,2]. This method involves drilling and the subsequent detonation of explosives, which fracture the targeted rock mass and transmit a significant amount of energy to the surrounding rock formations. As a result, these adjacent rock masses suffer varying degrees of damage, leading to a reduction in their mechanical properties and bearing capacity [3–5].

To achieve an optimal excavation profile and minimize the damage inflicted on the surrounding rock mass by blasting loads, plane blasting is routinely employed in the construction of roadways and tunnels [6,7]. A prevalent technique within this method is uncoupled charging, which, compared to coupled charging, greatly reduces the damage to the rock adjacent to the borehole. Traditionally, uncoupled charging has been assumed to involve a concentric setup, where the center of the drill hole aligns precisely with the center of the explosive charge. However, practical engineering demands often dictate the drilling of holes at specific angles, leading to either horizontal or vertical inclinations. Due to gravitational effects, the explosive material tends to settle on one side of the drill hole, challenging the achievement of a true concentric uncoupled configuration. Consequently, an eccentric uncoupled charging modality emerges [8]. In such cases, the energy from the explosive detonation impacts the surrounding rock mass unevenly, creating significant discrepancies in both the explosion stress field (SF) and the resultant blasting damage. This asymmetry is particularly notable in smooth blasting and presplit blasting operations, where it can cause substantial damage to the rock mass on the side intended to be preserved [9].

Currently, an extensive array of research has been conducted on rock damage induced by blasting, utilizing a variety of methodologies. Theoretical analyses [10,11], experimental investigations [12], and numerical simulations [13–15] have all contributed significantly to enhancing our comprehension of the mechanisms underlying rock breakage. More specifically, these studies have extensively examined the propagation characteristics of stress fractures caused by blasting in rocks, the attributes of rock damage, the crack propagation process, and the dynamic effects associated with rock breakage [16–20]. For example, Wang et al. [21] conducted an uncoupled blasting test on sandstone and employed computed tomography (CT) technology to quantitatively assess the extent of rock damage. Their results demonstrated that the level of damage to the rock mass decreases as the uncoupling coefficient increases. Similarly, Yang et al. [22] explored how the uncoupling coefficient influences stress evolution during uncoupled charge (UC) blasting. Wang et al. [23] implemented the circumferential water coupling blasting technique to overcome problems such as low signal-to-noise ratios and inaccurate seismic data results, particularly under challenging terrain conditions like those encountered in loess areas. Additionally, Fan et al. [24] provided a theoretical explanation of the rock fracture mechanism associated with single-hole blasting and modeled the variations in peak blasting pressure on the hole wall, as well as the dimensions of the crushing zone and the fracture zone, in relation to the uncoupling coefficient. However, in the aforementioned studies on UC blasting, whether through theoretical analysis, numerical simulation, or experimental testing, explosives and boreholes have consistently been treated as concentric charges. This standard assumption does not reflect the actual charge configurations commonly seen in real-world engineering projects. In practical engineering applications, due to gravitational effects, explosives often adhere to one side of the borehole, resulting in a predominantly eccentric uncoupled charge structure [16,17].

In the detailed study of eccentric UC blasting, Zhang Xiantang et al. [25], based on the combined action theory of stress waves and explosion gases, derived a calculation method for borehole spacing in smooth blasting with eccentric decoupled charges through theoretical analysis. They examined the positions of connecting cracks under different borehole spacing conditions and validated their findings using model tests and numerical simulations. Regarding air-coupled media, Jin Yang et al. [26] demonstrated that under eccentric charge conditions, the difference in peak wall pressure between the coupled side and the decoupled side of the charge section increases with the decoupling coefficient. The peak wall pressure on the coupled side was 4–11 times higher than that on the decoupled side, and a clear “eccentric effect” was observed in the damage zones of both the charged and uncharged sections. In a distinct approach, Pu Chuanjin [9] accurately measured the strain peaks on both sides of an eccentric UC model to clarify the eccentric characteristics of the SF distribution. He also performed experiments to determine the rate of sound velocity reduction in the surrounding rock before and after blasting. For water-coupled media, Wang Yanbing et al. [27] found that the water coupling coefficient and coupling form significantly influence the efficiency of explosion energy transmission, with an optimal decoupling coefficient identified. Eccentric water-coupled charges produce a distinct eccentric stress field, substantially increasing the peak wall pressure on the coupled side. WANG et al. [28] showed that eccentric decoupled charging generates noticeable asymmetry in pressure propagation. The coupled side exhibits higher peak pressure and faster loading rates, while the decoupled side experiences delayed wave arrival and lower peak pressure. This asymmetry intensifies with increasing decoupling and eccentricity coefficients. CHI et al. [29] indicated that decoupled charging effectively modulates the peak pressure and duration of blast stress waves, thereby influencing rock fragmentation and damage distribution. The decoupling ratio directly affects borehole wall pressure distribution and the extent of rock damage. As the decoupling ratio increases, borehole wall pressure decays exponentially, while the loading duration extends linearly.

Currently, extensive research is being conducted on rock damage induced by blasting, integrating theoretical analysis, experimental approaches, and numerical simulations. This body of work primarily examines the propagation characteristics of stress waves, traits of rock damage, the crack propagation process, and the dynamic effects of rock fragmentation. It is important to note that previous studies on UC blasting often assumed that the explosive was concentrically aligned within the borehole, which does not accurately reflect the typical conditions of eccentric UCs in real-world engineering projects. Although some studies have addressed eccentric UC blasting, most have focused on scenarios involving a single cartridge and have predominantly relied on model tests. There is a notable lack of comprehensive numerical simulations and in-depth research into configurations involving multiple cartridges and various angles. As such, in this study, the ANSYS/LS-DYNA nonlinear dynamic analysis platform is utilized to simulate various blasting conditions characterized by different charge quantities and structures. This approach allows for a comparative and analytical examination of the effects on hole wall pressure and damage under different charge configurations, ultimately aiming to provide a robust foundation for mitigating adverse impacts in actual blasting engineering applications.

## 2 Theoretical analysis

In applications such as roadway excavation, roof precracking, and bottom breaking processes, the implementation of the UC technique is utilized with the objective of minimizing excessive fragmentation of the borehole wall and optimizing the utilization of explosive energy. This technique involves an air gap between the explosive and the borehole wall, which provides space for the expansion of the explosive products. Consequently, when the mass of the explosive is held constant, the pressure exerted by the UC on the borehole wall is significantly lower compared to that exerted by a coupled charge. This reduction in pressure is pivotal in preserving the integrity of the borehole wall and enhancing the overall efficiency of the blasting operation.

**Fig 1:**
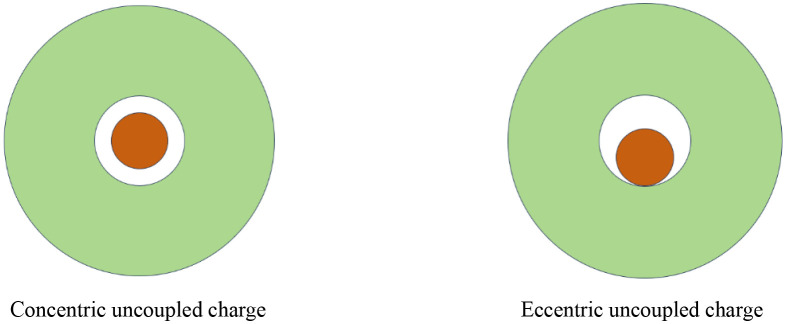
Uncoupled charge structure.

The UC is classified into two types: the concentric UC and the eccentric UC, distinguished by whether the center of the explosive aligns with the borehole center. Fig 1 displays these two types of UC configurations. Upon detonation of the explosive in these configurations, the blast wave propagates outward from the borehole wall toward the surrounding rock. The intensity of the blast wave diminishes rapidly with increasing distance from the borehole wall. The rock in close proximity to the borehole wall undergoes deformation due to the shock wave load, which exceeds its dynamic compressive strength. As the shock wave reaches the outer boundary of the crushing zone, it transitions into a stress wave, and its intensity falls below the dynamic compressive strength of the rock, ceasing to cause compressive failure. However, the radial compression of the rock induces circumferential tensile stresses. When these stresses exceed the dynamic tensile strength of the rock, radial cracks develop, leading to the formation of fracture zones. For the concentric UC, the pressure exerted by the detonation products on the borehole wall is relatively uniform. Conversely, in the case of the eccentric UC, the rock on the coupled side is initially impacted by the detonation products, with the pressure on the borehole wall gradually transmitted from the coupled side to the uncoupled side, resulting in a non-uniform pressure distribution around the borehole wall.

Under a coupled charge structure, the peak blasting load on the borehole wall can be determined by Equation (1) [30]:


$$P_{k}^{\prime }=\frac{\text{1}}{\text{1}+\gamma }\rho_{\text{1}}D_{e}^{\text{2}}$$ (1)

Where: *P*’_k_ is the peak value of the blasting load on the borehole wall during coupled charge blasting; *ρ*_1_ is the explosive density; *D*_e_ is the explosive detonation speed; *γ* is the isentropic adiabatic index of the detonation product, *γ* = 3.

For concentric radial UC blasting, the peak value of the blasting load on the borehole wall can be calculated using Equation (2) [31]:


$$P_{k}=\frac{\text{1}}{\text{2}}P_{k}^{\prime }\beta^{-\text{2}\gamma }n$$ (2)

Where: *P*_k_ is the peak value of the blasting load on the borehole wall during UC blasting; *β* is the radial uncoupling coefficient, *β* = *D*/*D*r, where *D* is the diameter of the borehole and *D*r is the diameter of the cartridge; *n* is the pressure enhancement coefficient, typically ranging from 8 to 11, with a general value of 8.

According to the attenuation law of stress waves in rocks, the equations governing the attenuation of peak radial and tangential stresses at any point within the rock mass can be expressed as follows [31]:


$$\sigma_{r}=P_{k}{\left(\overset{―}{r}\right)}^{-\alpha }$$ (3)

$$\sigma_{\theta }=-b\sigma_{r}$$ (4)

Where *σ*_r_ and *σ*_*θ*_ represent the peak values of radial and circumferential stresses respectively; *_r* denotes the specific distance, defined as *_r* = 2*r*_0_/*D*, where *r*_0_ is the distance from the calculation point to the cha*r*ge center; *α* is the attenuation coefficient; *b* is the lateral stress coefficient, calculated by *b* = *μ*_b_/(1-*μ*_b_), with *μ*_b_ = 0.8*μ* in engineering blasting applications, where *μ* is the rock’s Poisson ratio.

The stress at any point in the rock is given by:


$$\sigma_{i}=\frac{\text{1}}{\sqrt{\text{2}}}{\left[{\left(\sigma_{r}-\sigma_{\theta }\right)}^{\text{2}}+{\left(\sigma_{\theta }-\sigma_{z}\right)}^{\text{2}}+{\left(\sigma_{r}-\sigma_{z}\right)}^{\text{2}}\right]}^{\frac{\text{1}}{\text{2}}}$$ (5)

For ease of analysis and calculation, this scenario can be simplified to a plane strain problem [32]:


$$\sigma_{z}=\mu_{b}\left(\sigma_{r}+\sigma_{\theta }\right)=\mu_{b}(\text{1}-b)\sigma_{r}$$ (6)

By substituting Equation (6) and Equation (4) into Equation (4), the following is obtained:


$$\sigma_{i}=\frac{\text{1}}{\sqrt{\text{2}}}\sigma_{r}{\left[(\text{1}+b{)}^{\text{2}}-\text{2}\mu_{b}(\text{1}-b{)}^{\text{2}}\left(\text{1}-\mu_{b}\right)+\left(\text{1}+b^{\text{2}}\right)\right]}^{\frac{\text{1}}{\text{2}}}$$ (7)

Where *σ*_i_ represents the magnitude of stress at any point in the rock.

According to the Mises criterion and the definition of damage, rock failure occurs if *σ*_i_ satisfies the following condition (8):



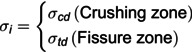

(8)


Where *σ*_*cd*_ and *σ*_*td*_ are the dynamic compressive strength and dynamic tensile strength of the rock, respectively.

When the stress wave propagates within the crushing zone, the attenuation coefficient is given by:


$$\alpha_{\text{1}}=\text{2}+\frac{\mu_{b}}{\text{1}-\mu_{b}}$$ (9)

By substituting Equation (2), Equation (3), and Equation (9) into Equation (7) and applying the criterion of Equation (8), the diameter of the crushing zone can be derived:


$$D_{s}=D{\left(\frac{\rho_{\text{1}}D_{e}^{\text{2}}\beta^{-\text{2}\gamma }nO}{\text{2}\sqrt{\text{2}}\sigma_{cd}(\gamma +\text{1})}\right)}^{\frac{\text{1}}{\alpha_{\text{1}}}}$$ (10)

Where *D*_s_ is the diameter of the crushing zone;


$$O={\left[(\text{1}+b{)}^{\text{2}}+\left(\text{1}+b^{\text{2}}\right)-\text{2}\mu_{b}\left(\text{1}-\text{2}\mu_{b}\right)(\text{1}-b{)}^{\text{2}}\right]}^{\frac{\text{1}}{\text{2}}}$$

At the boundary between the crushing zone and the crack zone, the following can be determined using Equations (7) and (8):


$$\sigma_{R}=\sigma_{r}|r=r_{s}=\frac{\sqrt{\text{2}}\sigma_{cd}}{O}\;$$ (11)

Where *σ*_*R*_ indicates the stress at the interface between the crushing zone and the crack zone.

Outside the crushing zone, the stress wave continues to propagate peripherally, and the attenuation coefficient is defined by:


$$\alpha_{\text{2}}=\text{2}-\frac{\mu_{b}}{\text{1}-\mu_{b}}$$ (12)

Using Equations (3), (7), (8), and (10)–(12), the diameter of the fracture region resulting from UC blasting can be calculated:


$$D_{T}={\left(\frac{\sigma_{R}O}{\sqrt{\text{2}}\sigma_{td}}\right)}^{\frac{\text{1}}{\alpha_{\text{2}}}}{\left(\frac{\rho_{\text{1}}D_{e}^{\text{2}}\beta^{-\text{2}\gamma }nO}{\text{2}\sqrt{\text{2}}\sigma_{cd}(\gamma +\text{1})}\right)}^{\frac{\text{1}}{\alpha_{\text{1}}}}D$$ (13)

Where *D*_T_ indicates the diameter of the fracture region.

## 3 Numerical simulation process

### 3.1 Numerical model

**Fig 2:**
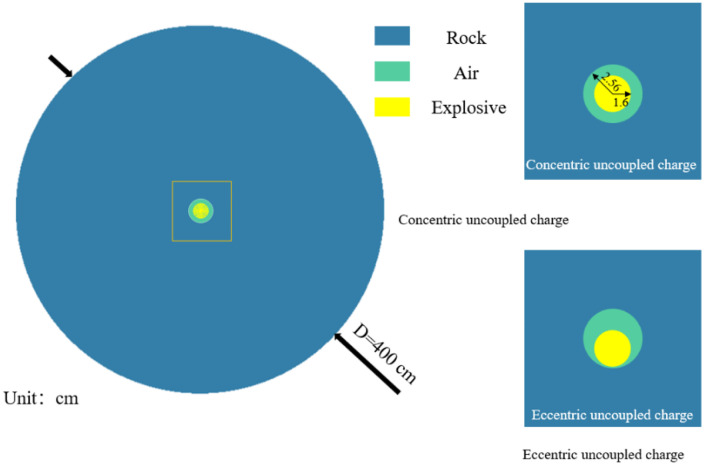
Numerical model.

The effects of blasting are investigated using a single-hole blasting model. Upon detonation of the explosive in the rock, notable deformation and potential fracturing of the rock mass occur. Within the framework of ANSYS/LS-DYNA numerical simulation software, the Arbitrary Lagrangian-Eulerian (ALE) algorithm is utilized. In this model, the Lagrange algorithm is applied to the rock and the Euler algorithm to both the air and the explosive. The entire model adopts a cm-g-us unit system and establishes a plane computational model. Symmetric constraints are imposed on the nodes of the symmetric plane, and non-reflective boundaries are applied to all four sides of the model. The model’s diameter measures 400 cm, with a thickness of 2 cm. The cartridge has a diameter of 3.2 cm, and the borehole diameter is 5.12 cm, resulting in an uncoupling coefficient of 1.6. Fig 2 visually represents the model. The initial volume fraction method is employed to optimize the positioning and sizing of the explosive during eccentric charging, enhancing both the modeling process and solution accuracy compared to traditional multi-substance fluid-structure coupling algorithms [33].

### 3.2 Materials and parameters

(1) Rocks

Rock materials are classified as porous, brittle substances. When exposed to external forces, pre-existing fissures within these materials begin to expand and interconnect, eventually leading to the creation of extensive macroscopic fractures. This progression ultimately results in the degradation and potential failure of the material. The fracture development in rock materials typically progresses through several distinct stages, including elastic mechanics, fracture mechanics, and damage mechanics [34]. The RHT model [35] is a comprehensive tensile and compressive damage model that considers the effects of failure strength of rock materials under conditions of blasting and dynamic loading. This model accounts for various factors including damage softening, strain hardening, strain rate, and impact pressure. Wang et al. [36] found that the RHT model is highly effective for simulating the blasting behavior of rock materials. In this study, the RHT model is utilized for numerical simulations with granite as the specific rock material under examination.

**Fig 3:**
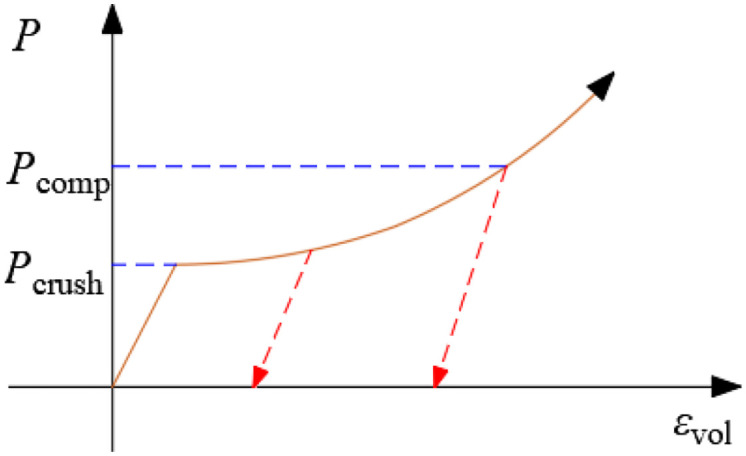
*P-α* State equation.

Given the porous nature of brittle materials like rocks, their mechanical behavior under different states is characterized using the p-α state equation within the RHT model, illustrated in Fig 3.

Within the RHT model, the damage evolution equation is defined as:


$$\text{0}\leq D=\sum \frac{\Delta \varepsilon_{p}}{\varepsilon_{p}^{f}}\leq \text{1}$$ (14)

$$\varepsilon_{p}^{f}={\left[p^{*}-(\text{1}-D)p_{t}^{*}\right]}^{D_{\text{2}}}\geq \varepsilon_{f,min}$$ (15)

Where *D* represents the damage variable; △*ε*_p_ denotes cumulative plastic deformation; *ε*^f^_p_ is the failure plastic strain; P^*^ signifies the standardized failure pressure; *ε*_*f*_,_min_ is the minimum allowable plastic strain; *D*_1_ and *D*_2_ are constants. The damage variable, *D*, ranges from 0 to 1, where higher values indicate increased material damage. The parameters for the rock RHT model used in this study are referenced from [35], with specific values listed in Table 1.

**Table 1:** RHT parameters of rocks.

Parameter	Value	Parameter	Value
Mass density RO (kg/m^3^)	2660	Break compressive strain rate EC	3E + 25
Elastic shear modulus SHEAR(GPa)	21.9	Break tensile strain rate ET	3E + 25
Relative shear strength FS^*^	0.18	Lode angle dependence factor Q_0_	0.68
Relative tensile strength FT^*^	0.04	Lode angle dependence factor B	0.01
Parameter for polynomial EOS T_1_ (GPa)	35.27	Compressive yield surface parameter GC^*^	0.53
Parameter for polynomial EOS T_2_ (GPa)	0	Tensile yield surface parameter GT^*^	0.7
Damage parameter D_1_	0.04	Crush pressure PEL (MPa)	125
Damage parameter D_2_	1.0	Compaction pressure PCO (GPa)	6
Hugoniot polynomial coefficient A_1_ (GPa)	35.27	Shear modulus reduction factor XI	0.5
Hugoniot polynomial coefficient A_2_ (GPa)	39.58	Eroding plastic strain EPSF	2.0
Hugoniot polynomial coefficient A_3_ (GPa)	9.04	Minimum damaged residual strain EPM	0.01
Failure surface parameter A	1.60	Porosity exponent NP	3.0
Failure surface parameter N	0.61	Initial porosity ALPHA	1.0
Residual surface parameter AF	1.60	Pressure influence on plastic flow in tension PTF	0.001
Residual surface parameter NF	0.61	Tensile strain rate dependence exponent BETAT	0.036
Parameter for polynomial EOS B_0_	1.22	Compressive strength FC (MPa)	167.8
Parameter for polynomial EOS B_1_	1.22	Compressive strain rate dependence exponent BETAC	0.032
Reference compressive strain rate EOC	3E-05	Gruneisen gamma GAMMA	0
Reference tensile strain rate EOT	3E-06

(2) Explosives

In the ANSYS/LS-DYNA software, the high-performance explosive material model *MAT_HIGH_EXPLOSIVE_BURN is used alongside the JWL state equation to describe the volume, pressure, and energy characteristics of the explosive products during detonation. The formula employed is as follows [37]:


$$P=A\left(\text{1}-\frac{\omega }{R_{\text{1}}V}\right)exp\left(-R_{\text{1}}V\right)+B\left(\text{1}-\frac{\omega }{R_{\text{2}}V}\right)exp\left(-R_{\text{2}}V\right)+\frac{\omega E_{\text{0}}}{V}$$

Where *P* is the detonation pressure; *V* represents the relative volume; *E*_0_ is the initial specific internal energy; *A*, *B*, *R*_1_, *R*_2_, *ω* are material constants, detailed in Table 2.

**Table 2:** The parameters of explosive.

Density /(kg/m^3^)	Detonation velocity (m/s)	*P*cj/GPa	*A*/GPa	*B*/GPa	*R*_1_	*R*_2_	ω	*E*_0_/GPa
1320	6690	16	586	21.6	5.81	1.77	0.282	7.38

(3) Air

The material model *MAT_NULL is adopted for air, with its state equation expressed by the linear polynomial *EOS_LINEAR_POLYNOMIAL:


$$P=C_{\text{0}}+C_{\text{1}}V+C_{\text{2}}V^{\text{2}}+C_{\text{3}}V^{\text{3}}+\left(C_{\text{4}}+C_{\text{5}}V+C_{\text{6}}V^{\text{2}}\right)E_{\text{0}}$$

In the equation, *C*_0_ through *C*_6_ are the relevant parameters, where *C*_4_ and *C*_5_ are set at 0.4, *E*_0_ = 2500 MJ/m^3^, *V* is normalized to 1.0, and the remaining parameters are zero.

### 3.3 Validation of the numerical model

**Fig 4:**
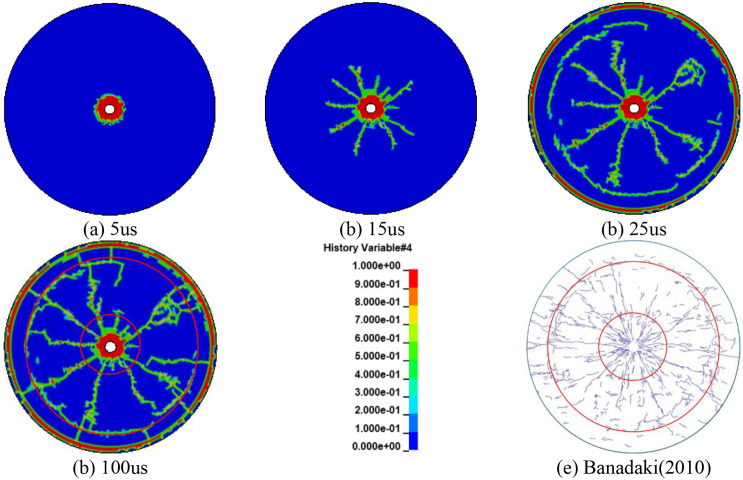
Comparison of numerical simulation results and experimental results.

To better verify the accuracy of the numerical results in this paper, the numerical simulation was compared with experimental results. The rock sample used was granite with a density of 2.66 g/cm³ and a Poisson’s ratio of 0.26. Fig 4a–d present the simulation results of single-hole blasting in rock using the calibrated RHT constitutive model implemented in ANSYS/LS-DYNA. Fig 4e shows the experimental results from Banadaki [38] on the propagation of blasting cracks in single-hole rock blasting. By comparing Fig 4d and 4e, it can be observed that a crushed zone and a fracture zone appear in Fig 4d, and the extents of these two zones are in good agreement with the experimental results in Fig 4e. This demonstrates the reliability of the numerical simulation and lays a foundation for subsequent simulations of blasting crack propagation.

## 4 Results and discussion

### 4.1 Characteristics of effective stress distribution

**Fig 5:**
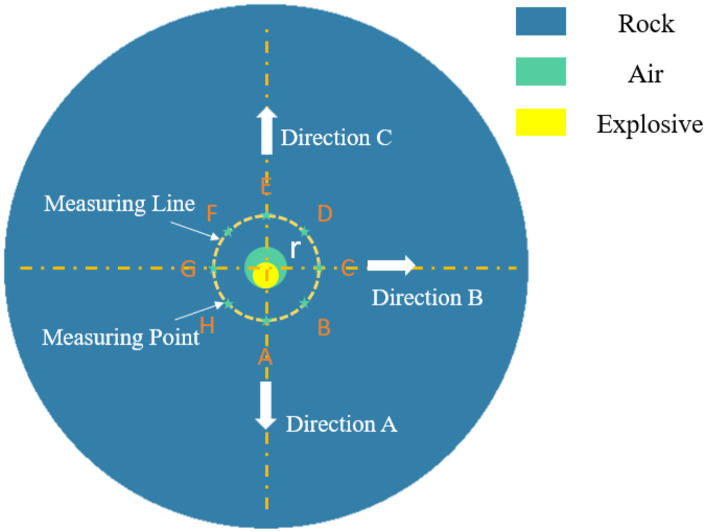
Location of monitoring points.

To examine the interaction between the detonation products and the surrounding rock near the borehole following the explosion, both the detonation products and explosive gases exert stress on the borehole walls. To thoroughly explore the pattern of stress distribution, and using a single cartridge as an example, measurement points at varying radii were selected along a circular measuring line at different distances from the borehole (Fig 5).

**Fig 6:**
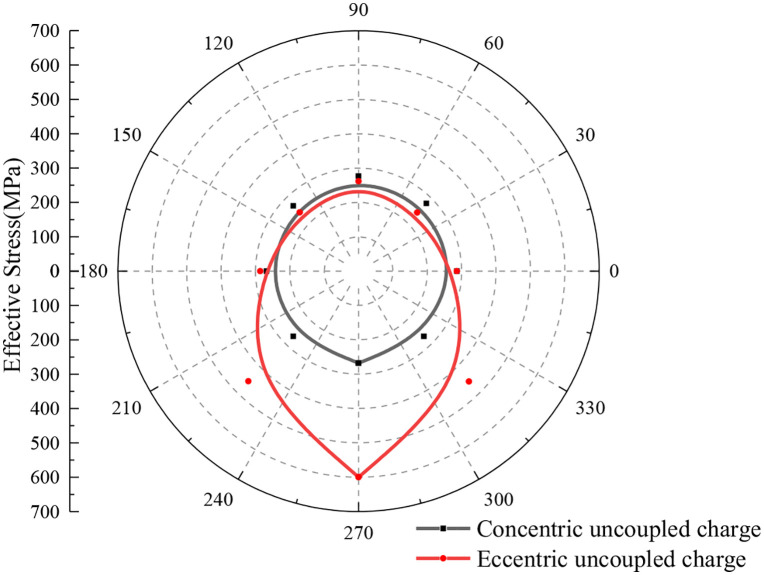
Effective stress distribution of concentric uncoupled and eccentric uncoupled charge blasting when r=6 cm (single cartridge).

Fig 6 displays the ES distribution in the rock around the borehole during the detonation of a single charge, induced by both concentric uncoupled and eccentric UC blasting. This figure provides a comparative analysis of the stress distributions in the surrounding rock under these two different charging conditions. For comparison purposes, a circular measuring line with a radius of 6 cm was used. As evident from the figure, when the concentric UC is situated equidistant from the explosive center, the effective stress (ES) in the rock remains relatively uniform, and its distribution closely resembles a circular pattern. This observation aligns with the experimental findings reported by Yang et al. [22], where the peak ES reached approximately 268 MPa.

The point of coupling between the charge position and the borehole wall is located at the lowest part of the borehole, and the model exhibits vertical symmetry. Notably, the two measurement points on the vertically symmetrical circular line display nearly identical ES values, with the distribution characteristics of ES on the left and right sides reflecting each other symmetrically. It is also apparent that the overall distribution of ES maintains the vertical symmetry of the model. From the figure, it is clear that the distribution of ES is markedly eccentric on the side coupled with the borehole. The ES at the bottom coupling point (CP) reaches its maximum, gradually diminishing from the bottom CP to the top uncoupling point. Specifically, the peak ES at the bottom CP is recorded at 599.89 MPa, while at the top uncoupling point, it is 261.63 MPa, indicating that the peak ES at the bottom is approximately 2.29 times that at the top.

In the case of eccentric UC, the ES on the coupled side exceeds that of the concentric UC at the same monitoring point. Compared to the concentric UC, the ES in the rock below the horizontal line of the borehole is notably higher, while that above the horizontal line is relatively lower. This difference suggests that the eccentric UC alters the transfer and distribution of explosive energy, which in turn influences the eccentric characteristics observed in the ES distribution of the rocks surrounding the borehole.

**Fig 7:**
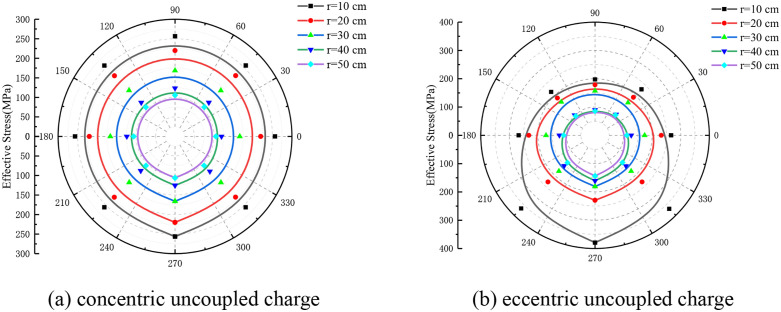
Effective stress on circular line with different r.

Fig 7 illustrates the distribution characteristics of rock ES along different radial measurement lines under both concentric and eccentric UC conditions. As shown in Fig 7a, there is a noticeable decrease in the ES of the rock as the radial distance increases. Additionally, the ES distribution approximates a circular shape and exhibits symmetry with respect to the model’s symmetry axis. Fig 7b reveals that under eccentric UC conditions, the ES distribution remains vertically symmetric across varying radii of the circular monitoring line. Furthermore, as the radius of the monitoring line increases, the ES at each monitoring point progressively declines, and the distribution assumes an eccentric pattern. The ES decreases progressively from the lower CP to the upper uncoupling monitoring point, indicating that the peak of maximum ES is located at the bottom coupling, while the minimum ES peak is at the top uncoupling. Additionally, as the radius of the circular monitoring line increases, the difference in ES along the same line becomes increasingly negligible. When the radius reaches 50 cm, the ES at the monitoring points is nearly uniform, and the distribution pattern closely resembles that observed under concentric UC conditions.

**Fig 8:**
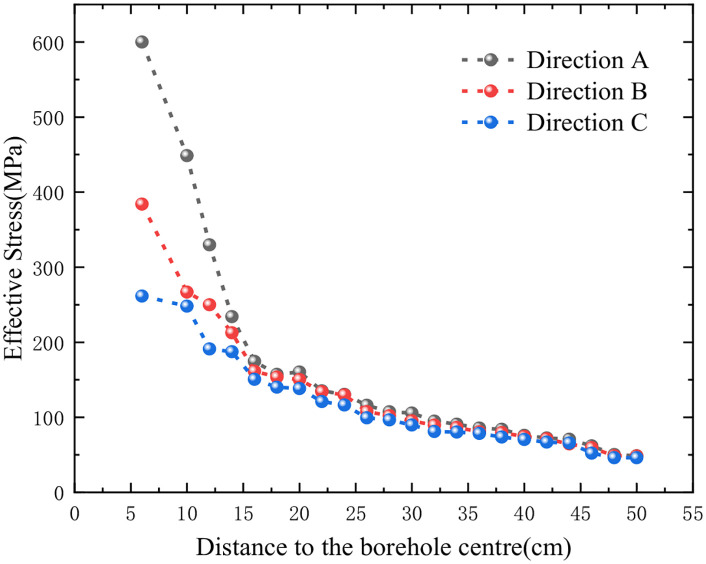
Effective stress attenuation in directions A, B and C (single cartridge).

Further investigation was conducted into the attenuation of ES with increasing distance from the borehole. Considering the relative positioning of the borehole and the charge, directions A, B, and C were selected for analysis (as illustrated in Fig 5). Fig 8 depicts the attenuation of ES in these directions. It is clear that at equal distances from the borehole center, the ES in direction A is higher than in direction B, with direction C exhibiting the lowest stress. As the distance from the borehole center increases, the ES in all three directions decreases, and the differences among them diminish. When the distance exceeds 50 cm, the ES curves in the three directions essentially converge. This observation suggests that the influence of the eccentric UC on ES attenuation is confined to within 50 cm from the borehole center. Beyond this range, the SF generated by eccentric UC blasting is comparable to that produced by concentric UC blasting.

**Fig 9:**
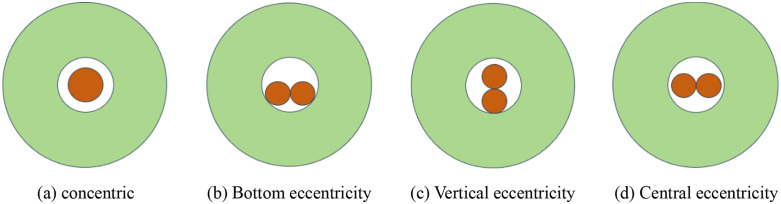
Different charge structures of double cartridge.

**Fig 10:**
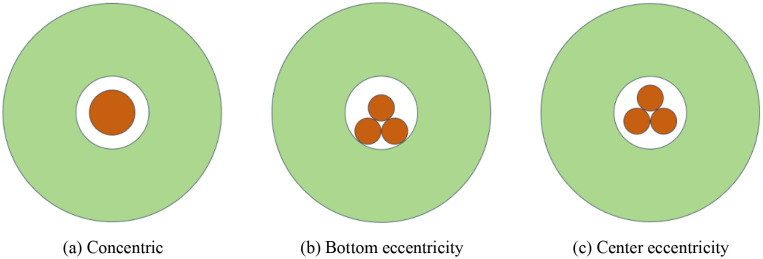
Different charge structures of the three rolls.

The content discussed herein pertains to the characteristics of ES distribution when a single cartridge is loaded into a borehole. In practical engineering applications, the dimensions of traditional Chinese medicine cartridges, which presumably refer to a specialized type of cartridge relevant to the context, cannot be modified arbitrarily. Consequently, in certain situations, it becomes necessary to enlarge the borehole diameter to accommodate the bundling of two or more cartridges, thereby meeting the engineering requirements. Due to gravitational forces acting on the cartridges, they tend to move closer to the borehole wall unless preventative measures are taken. To investigate the characteristics of ES distribution in scenarios involving multiple cartridges, studies typically focus on configurations with two or three cartridges. In these studies, the uncoupling coefficient and the mass of each cartridge are held constant. This setup results in the formation of multiple angular charge structures, as depicted in Figs 9 and 10.

**Fig 11:**
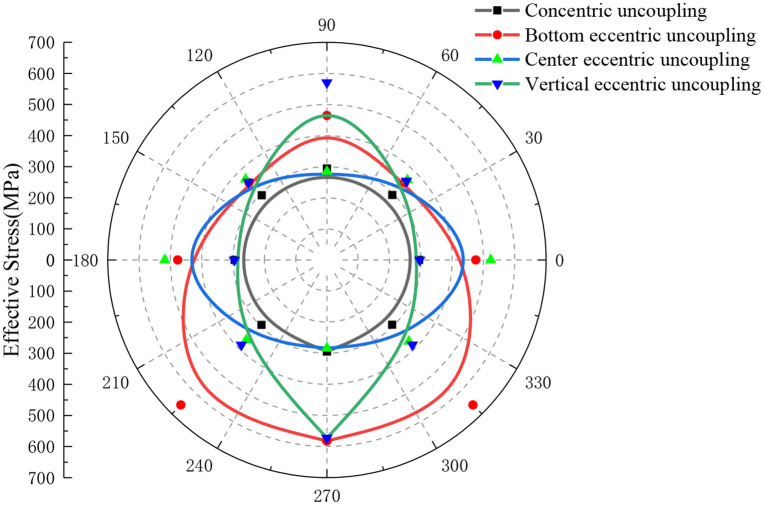
Effective stress distribution of concentric uncoupled and eccentric uncoupled charge blasting when r=6 cm (double cartridge).

In this study, the effects of charge configuration on the distribution of ES in blasting operations are analyzed. The experimental setup involves maintaining a constant cartridge mass and a fixed distance from the borehole center. Notably, the analysis compares the stress distribution characteristics between concentric UC and eccentric UC configurations, as illustrated in Fig 11. It is observed that with concentric uncoupled charges, when the distance to the monitoring point from the borehole’s center remains unchanged, the ES values are almost identical, exhibiting a nearly circular distribution pattern. The average ES in this configuration is approximately 294 MPa. Conversely, with eccentric uncoupled charges, particularly when employing a double-coil bottom configuration, the mean ES peaks at a significantly higher value. This distribution demonstrates vertical symmetry, where monitoring points on the same vertically symmetrical line experience nearly identical ESs. The maximum ES recorded near the bottom coupling reaches up to 659 MPa, which is approximately 1.88 times the ES of 351 MPa observed at the nearest point on both sides of the top uncoupled vertex. In situations where the double cartridge is uncoupled in the middle, the closest horizontal monitoring point to the cartridge records a maximum ES of 523 MPa, approximately 1.85 times that of the 283 MPa measured at the vertically farthest point. Here, the ES distribution assumes a horizontal elliptical shape. Similarly, when the dual cartridge is uncoupled vertically, the maximum ES at the lower CP in the vertical direction registers 573 MPa, about 1.94 times the stress at the most distant horizontal monitoring point from the borehole’s center. The ES at the upper uncoupled test point measures 570 MPa, highlighting a minimal variation due to proximity to the borehole, thus forming a vertical elliptical distribution.

Overall, the results clearly indicate that the ES associated with a double charge configuration significantly surpasses that of a single charge. Among the tested configurations, the eccentric UC at the bottom demonstrates the highest ES. The ES values at both horizontal and vertical monitoring points are comparable to those observed in central and vertically eccentric uncoupled charges. Importantly, the bottom coupling measuring point consistently shows higher stress levels compared to the same points under other charge configurations. These findings suggest that the rock-breaking efficacy of a double charge is superior to that of a single charge, particularly when the charge is eccentrically uncoupled at the bottom.

**Fig 12:**
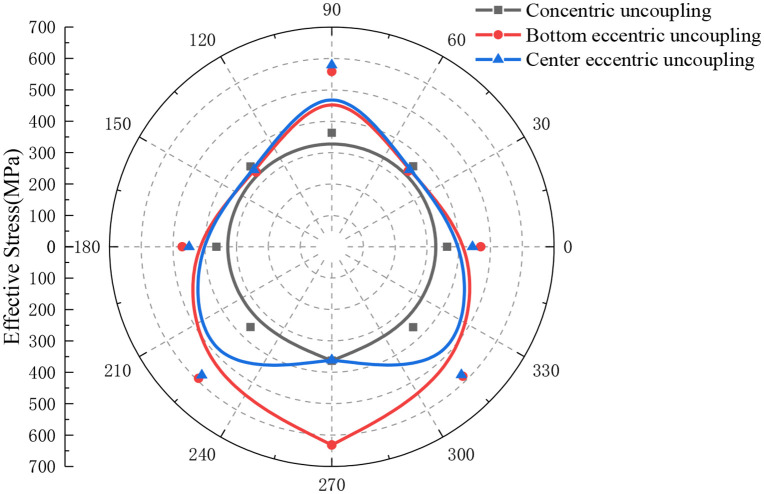
Effective stress distribution of concentric uncoupled and eccentric uncoupled charge blasting when r=6 cm (three charge rolls).

The distribution characteristics of ES at monitoring points under varying charge configurations for three rolls are depicted in Fig 12. When employing three charge rolls of identical mass configured in a concentric uncoupled arrangement, the ES values at a constant distance from the center of the blast hole are observed to be virtually identical. Here, the ES distribution closely resembles a circular pattern, with an average ES of approximately 363 MPa. In the scenario where a three-coil bottom eccentric UC is used, while maintaining consistent monitoring points, the average ES reaches a relatively higher peak. Furthermore, the distribution of ES in this case demonstrates vertical symmetry, where two points on a vertically symmetric monitoring line exhibit almost equal ES values. Notably, the maximum ES recorded at the bottom monitoring point A is 631 MPa, which is roughly 1.88 times higher than the ES of 336 MPa measured at the nearest points on either side at the top uncoupled vertex. When the middle section of the three charge rolls is uncoupled, monitoring points B, E, and H, which are relatively closer to the charge rolls, display a peak ES of approximately 578 MPa, with values being nearly the same across these points. Additionally, the ES distribution in this arrangement also shows vertical symmetry.

In conclusion, the data clearly indicate that the rock-breaking efficacy of the double roll configuration is superior to that of the single roll when both are of equivalent mass. Moreover, the rock-breaking effect achieved with the eccentric UC at the bottom proves to be more effective when compared to other configurations.

**Fig 13:**
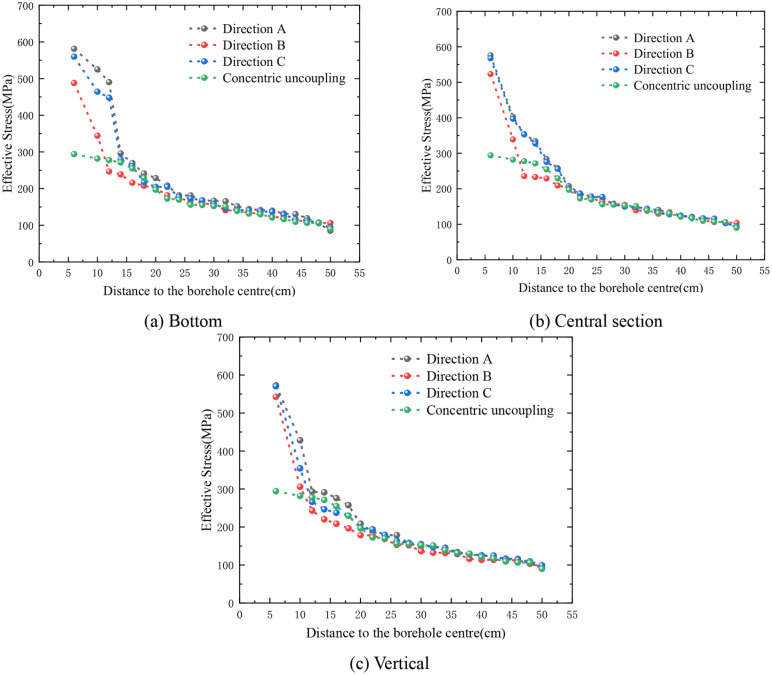
Effective stress attenuation in directions A, B and C (double cartridge).

The attenuation profile of the eccentric uncoupled ES at the base of the dual-cartridge system demonstrates that, at equal radial distances from the borehole’s center (as depicted in Fig 13a), the ES in Direction A exceeds that in Direction C, with the stress in Direction B being the third highest. Notably, prior to reaching a radial distance of 16 cm from the borehole’s center, the decrease in ES at the measurement site occurs most rapidly, subsequently transitioning to a more gradual rate of attenuation. Similarly, the ES attenuation profile for the eccentric UC positioned centrally within the double cartridge shows that the ESs in Directions A and C are nearly equivalent and both are higher than that in Direction B. Here, the attenuation rate of ES at the measurement point is highest within a 20 cm radius from the borehole’s center, beyond which it moderates (Fig 13b). For the vertically positioned eccentric UC within the double-cartridge configuration, the ES attenuation profile indicates that within a 20 cm radius from the borehole’s center, the ES in Direction A is greater than in Direction C, with the most rapid decay observed. Beyond this 20 cm radius, the ESs in Directions A and C nearly equalize, while the ES at monitoring point B remains the lowest at comparable distances (Fig 13c).

The graphical representation elucidates that as the distance from the borehole’s center increases, the ES decreases across all three directions, and the disparity between them gradually narrows. This observation suggests that the influence of the bottom eccentric charge (EC) on the SF is limited to within 24 cm of the central point of the blast hole. Similarly, the impact of the middle EC is confined to a 20 cm radius, and the effect of the vertical EC extends up to 36 cm from the center. Beyond these specified radii, the SF patterns resulting from EC blasting begin to resemble those produced by concentric charge blasting.

**Fig 14:**
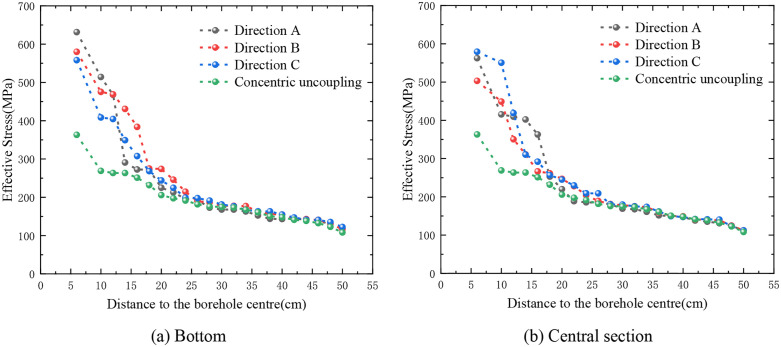
Effective stress attenuation in directions A, B and C (double cartridge).

The attenuation dynamics of ES for three-cartridge blasting within a single borehole along directions A, B, and C are depicted in Fig 14. The attenuation curve for the eccentric uncoupled ES at the base of the three-cartridge setup demonstrates superior performance compared to that of the concentric UC configuration. Initially, within the first 12 cm, the ES in direction A is higher than in directions B and C. Then, from 12 cm to 24 cm, the ES in direction B surpasses those in directions A and C. Beyond a radial distance of 24 cm, the ES values in all three directions converge towards those observed in the concentric UC structure (as illustrated in Fig 14a).

The decay curve for the ES at the eccentric uncoupled central segment of the three cartridges shows that the ES in all three directions exceeds that of the concentric uncoupled structure. Notably, the stress in directions A and C displays significant fluctuations, whereas the stress in direction B exhibits a more consistent variation (Fig 14b). Beyond 28 cm, the ES values in the three directions become virtually indistinguishable from those associated with the concentric UC structure.

From these observations, it is apparent that the influence of the bottom EC on the SF is confined to within 24 cm from the center of the blasting hole. Likewise, the impact of the middle EC extends to 28 cm from the center, with only a slight difference in the affected ranges. Beyond these specified distances, the SF patterns resulting from EC blasting closely approximate those generated by concentric charge blasting.

### 4.2 Damage characteristics of borehole surrounding rock

**Fig 15:**
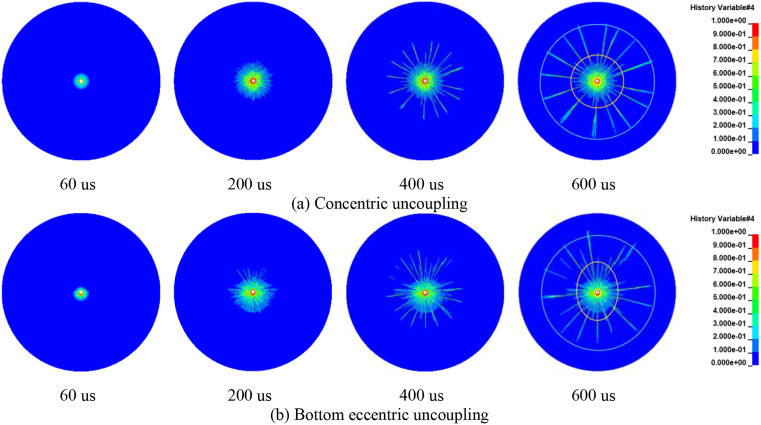
Damage evolution process of concentric and eccentric uncoupled blasting rock (single cartridge).

Using single-charge roll blasting as a case study, this section illustrates the progressive evolution of rock damage within the borehole influenced by both concentric and eccentric uncoupled charging methods. For this analysis, a rock area with a radius of 200 cm was selected, as depicted in Fig 15. The RHT constitutive model was employed to simulate the development of cracks, represented by damage contours ranging from 0 to 1. In these simulations, the color blue indicates a contour level of 0, signifying the rock’s elastic state where it remains intact and undeformed. Conversely, the color red represents a level 1 isoline, indicative of complete rock failure, characterized by a loss of structural integrity. Intermediate colors between these extremes correspond to various isolines, each representing different degrees of damage sustained by the rock.

The stress distribution in the rock mass after explosive blasting, considered alongside the dynamic compressive strength of the rock [39,40], enables classification of the damage zones. Regions with a damage factor exceeding 0.9 are defined as the crushing zone, where the rock is pulverized and fragmented due to intense forces. Areas where the damage factor ranges from 0.1 to 0.9 constitute the fracture zone, characterized by the formation and propagation of cracks that weaken the rock structure. Regions with a damage factor between 0 and 0.1 are identified as the elastic zone, where the rock retains its elastic properties and has sustained minimal damage.

Fig 15a clearly shows that with the application of a concentric UC, crack propagation is uniform and radiates outward from the hole. The edges of the cracks extend and interconnect, maintaining an approximately circular shape, and the distribution of crack growth exhibits symmetry along both horizontal and vertical axes. At 60 μs, a distinct crushing zone is visible, marked by the outward propagation of the blasting shock wave. By 200 μs, a dense crack zone has formed, with cracks continuing to expand and leading to a sparse crack zone by 600 μs. This temporal progression allows for a comprehensive visualization of the crack growth resulting from the blast.

When utilizing a bottom eccentric UC, as illustrated in Fig 15b, the crack growth exhibits a dominant direction. The density of cracks is considerably higher on the coupled side, where the explosive directly interfaces with the borehole wall. Throughout the period from 60 μs to 600 μs, which captures the progression of rock crack growth, the crack development on the coupled side is notably more pronounced and numerous. This observation aligns with the findings of Zong et al. [41], which suggest that the damage distribution on the coupled side is substantially greater than on the uncoupled side. Unlike the concentric UC, the crack distribution here is symmetric only along the vertical axis, which also acts as the symmetry axis in the numerical simulation. Clearly, the bottom eccentric UC directs a greater amount of explosive energy towards the side coupled with the hole wall, thereby intensifying the damage to the rock.

**Fig 16:**
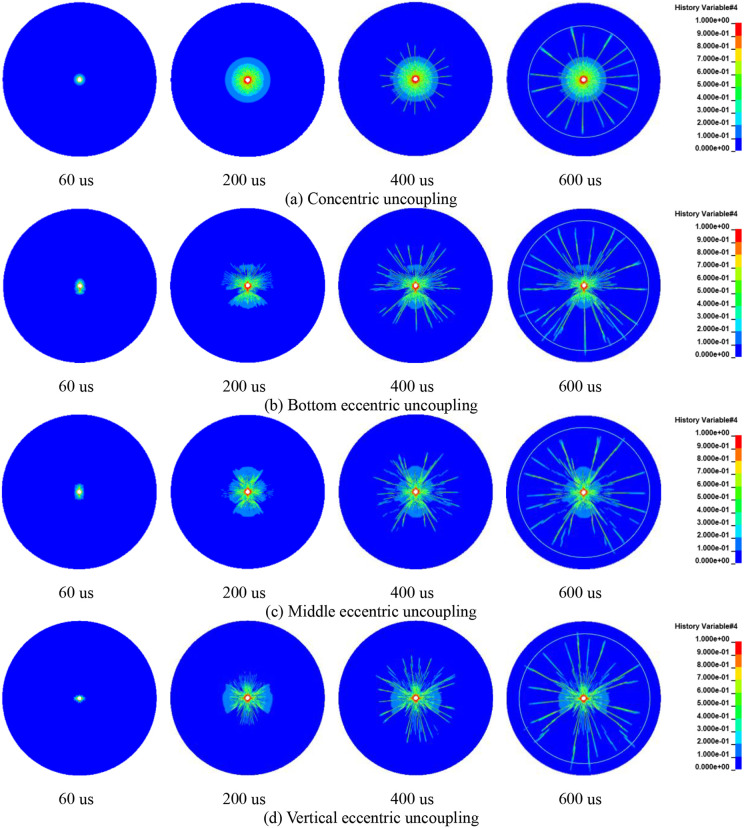
Damage evolution process of concentric and eccentric uncoupled blasting rock (double cartridge).

Fig 16 delineates the evolution of rock blasting damage under conditions of identical cartridge mass but differing charging structures. As evident from the figure, there exists a clear correlation between the extent of rock damage and the positioning of the charge. The crack growth distribution is closely related to the numerical model, with the symmetry axis of the model aligning precisely with that of the crack distribution. When the charge is configured in a concentric uncoupled manner, the cracks radiate outward uniformly around the borehole. At all stages, the connecting lines of the cracks maintain an approximately circular shape, and the crack distribution exhibits symmetry with respect to both the horizontal and vertical axes. In contrast, with bottom eccentric uncoupled charging, the crack pattern is irregular, initially manifesting on the coupled side where the density of cracks is significantly high. The crack distribution demonstrates symmetry solely around the vertical axis. With the middle portion of the charge uncoupled, the cracks initially produce a dominant vertical crack. Subsequently, the crack distribution evolves towards symmetry about both the horizontal and vertical axes. When employing a vertical UC, a dominant horizontal crack forms between the two charge rolls. As the cracks continue to expand, the crack distribution achieves symmetry around the vertical axis, with the density of cracks on the single coupled side being comparatively higher.

**Fig 17:**
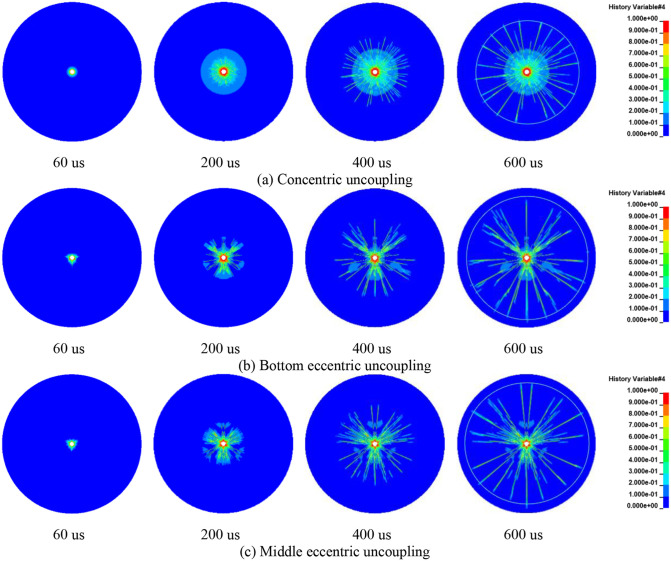
Damage evolution process of concentric and eccentric uncoupled blasting rock (three charge volumes).

Under conditions of identical cartridge mass, the evolution of rock damage for the three-cartridge configuration with diverse charging structures is depicted in Fig 17. When the charge is set up in a concentric uncoupled manner, the crack propagation behavior consistently aligns with previous observations. Specifically, the connecting lines of the cracks maintain an approximately circular shape throughout the process, and the crack distribution exhibits symmetry with respect to both the horizontal and vertical axes. In configurations where either the bottom eccentric UC or the middle eccentric UC is employed, the crack growth patterns show relative similarity. Immediately after blasting, crack initiation predominantly occurs in three leading directions. The state of rock damage at 200 μs is illustrated in Fig 17b and 17c. Notably, when employing the eccentric UC, the crack distribution displays symmetry around the vertical axis. Furthermore, the length of crack propagation exceeds that observed with the concentric UC. This suggests that the eccentric UC more effectively harnesses the explosive energy, facilitating more extensive rock crack propagation and, consequently, enabling improved utilization of the blasting energy.

**Fig 18:**
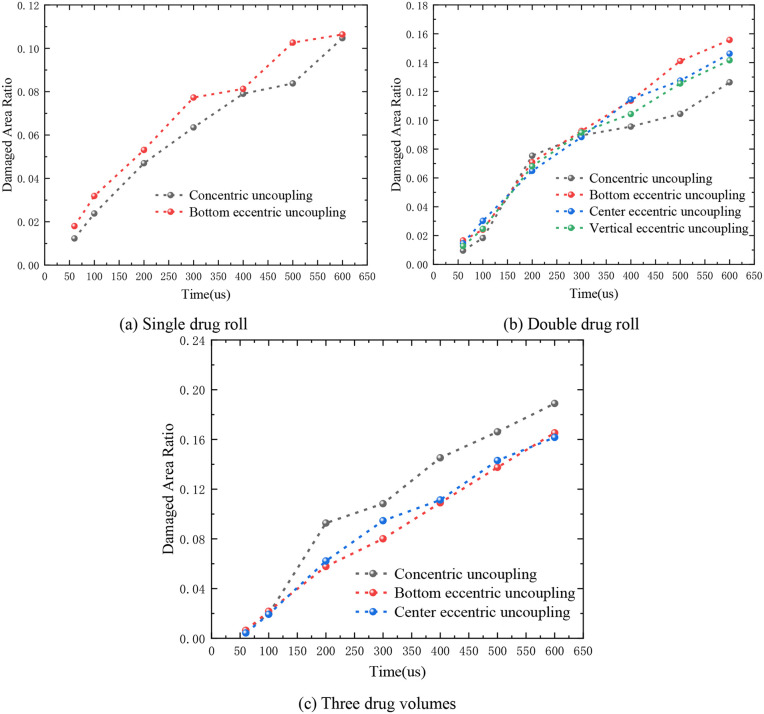
Damage degree of blasting rock with different charge structures of concentric uncoupling and eccentric uncoupling.

The analysis provided here offers primarily a qualitative elucidation of the rock damage; it does not quantitatively reflect the extent of damage to the rock. To address this limitation, the Damage Area Ratio (DAR) was introduced as a metric to quantify the degree of damage within a specified area. Fig 18 presents a comparison of the damage area ratios under the blasting impact of both concentric uncoupled and eccentric UCs, considering different charging structures within a study area with a radius of 200 cm.

In the case of the single-cartridge scenario, the variation in the damage area ratios between two distinct conditions was relatively minor during the same time interval. However, it is noteworthy that the damage area ratio for the bottom UC consistently exceeded that of the concentric UC, as depicted in Fig 18a. Despite variations in the relative positions of the hole and the cartridge, the total energy transferred by the explosion to the rock remained equivalent. The damage area ratio exhibited a continuous upward trend, peaking at 400 μs and 600 μs, where the differences between the two scenarios became minimal and nearly indistinguishable. At other times, the damage area ratio of the bottom uncoupled charge invariably surpassed that of the rock under concentric UC.

For the double-cartridge configuration, the damage area ratios under different charging structures-concentric uncoupling, bottom uncoupling, middle uncoupling, and vertical uncoupling-can be categorized distinctly. The growth of the damage area ratio could be broadly segmented into two phases. From 50 μs to 300 μs, the damage area ratios under the four uncoupling modes escalated at a relatively rapid pace, with negligible differences among them. Between 300 μs to 600 μs, although the damage area ratios continued to rise, the rate of increase decelerated. Notably, the damage area ratio under concentric UC blasting consistently remained lower than those of other charge structures, while the damage area ratio for the rock with the bottom UC was the highest, as shown in Fig 18b.

Regarding the scenario with three charge rolls, the augmented number of charge rolls led to a configuration that more closely approximated a circular shape, resulting in a more uniform distribution of damage across the rock, as illustrated in Fig 18c. Between 50 μs and 100 μs, the damage area ratios under different structures were essentially identical. From 100 μs to 600 μs, the damage area ratio curves for the bottom eccentric uncoupling and the middle eccentric uncoupling were nearly superimposed. In contrast, the damage area ratio for the rock under concentric uncoupled charging continued to climb and was consistently greater than those of the rocks under eccentric charging structures.

In the aforementioned research endeavor, the impact of diverse charging structures on rock damage was analyzed by comparing the ratio of the rock blasting damage area to the total study area. Notable differences were observed in the degree of damage to the rock mass between the concentric uncoupled and eccentric uncoupled charging structures. While certain disparities were not overly pronounced, it was evident that the crack density in the upper and lower portions of the rock around the borehole varied during the blasting process with the eccentric UC. Notably, the crack distribution exhibited symmetry along the vertical axis.

**Fig 19:**
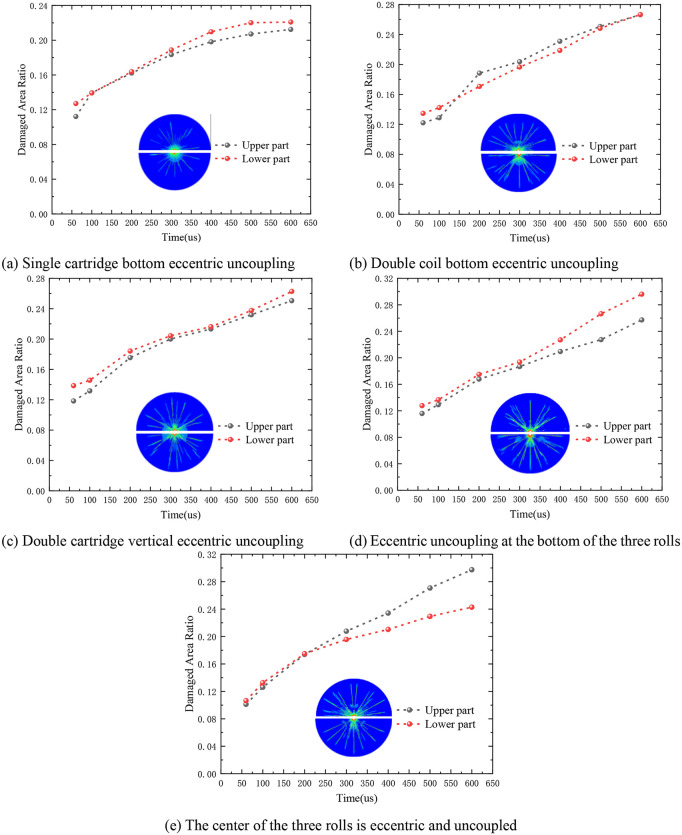
Comparison of damage degree in the upper and lower parts of the rock around the gun hole.

In this academic study, the rock in the vicinity of the borehole was segmented into upper and lower parts, with the damage area ratio computed independently for each segment. Fig 19 presents a comparative analysis of the damage area ratios between the upper and lower parts of the rock under different charging structures.

When employing a bottom eccentric uncoupled single cartridge, it is consistently observed that the damage area ratio in the lower part of the rock is higher compared to the upper part, as illustrated in Fig 19a. Over time, the damage areas for both the upper and lower parts increase. Notably, between 100 μs and 200 μs, the damage areas of the two parts were closely aligned. After 200 μs, the rate of increase in the damage area ratio began to decelerate. Initially, the damage in the lower part was more pronounced; however, as time progressed, the damage area of the upper part gradually exceeded that of the lower part, and interestingly, by 600 μs, the damage area ratios of the upper and lower parts equalized, as shown in Fig 19b.

From the data, it is evident that the damage area of the lower part invariably exceeds that of the upper part. Moreover, the damage areas of both the upper and lower parts grew in tandem over time, with their increasing trends nearly parallel. At 600 μs, the damage area of the lower part was approximately 1.05 times that of the upper part, as depicted in Fig 19c. In the case of the three-roll configuration, the damage area ratio of the lower part consistently surpassed that of the upper part. From 50 μs to 300 μs, the damage area ratios of both parts escalated steadily, with their growth curves aligning closely, as illustrated in Fig 19d. Post 300 μs, the damage area ratio of the lower part ascended at a faster pace than that of the upper part. Remarkably, at 600 μs, the damage area of the lower part was 1.15 times that of the upper part. Between 50 μs and 200 μs, the rock damage area ratios of the upper and lower parts were nearly indistinguishable, and the rate of increase was substantial. After 200 μs, the damage area ratio of the upper rock plateaued, while that of the lower rock continued to rise, albeit at a diminishing rate. Consequently, the disparity between the upper and lower rocks gradually widened, and astonishingly, at 600 μs, the damage area of the upper rock was 1.22 times that of the lower rock, as shown in Fig 19e.

In conclusion, it is evident that with identical charge mass but varying charging structures, significant discrepancies in the damage area ratio of the rock are observed. In practical engineering applications, efficient rock fragmentation can be achieved by precisely adjusting the number of charge rolls and optimizing their placement. For a single charge roll, the performance of the bottom eccentric UC is relatively outstanding. Specifically, compared to the concentric UC, the bottom eccentric UC, the middle eccentric UC, and the vertical eccentric UC all demonstrate enhanced effectiveness, among which the bottom eccentric UC exhibits the most preferable effects. Conversely, when employing three charge rolls, the concentric UC demonstrates a more favorable impact.

## 5 Conclusions

This study investigates the influence of different charge structures on blasting effects through theoretical analysis and fluid-solid coupling numerical simulations, focusing on the effective stress distribution and damage characteristics of the rock surrounding the borehole. The findings provide theoretical references and practical guidance for blasting engineering. The main conclusions are as follows:

(1) The stress field distribution under different charge structures exhibits clear eccentricity and limited influence range. With concentric decoupling charging, the blasting stress field is uniformly distributed, and the effective stress is essentially consistent at equal distances. In contrast, eccentric decoupling charging results in an asymmetric stress field, with significantly higher stress on the coupled side than on the decoupled side (ratio approximately 2.29:1). The influence range of eccentric charging is limited: within 50 cm for a single charge cartridge, 24 ~ 36 cm for two cartridges, and 24 ~ 28 cm for three cartridges. Beyond these ranges, the stress field distribution tends to align with that of concentric charging.
(2) The crack propagation pattern is closely related to the eccentricity of the charge. With concentric charging, cracks propagate uniformly outward from the borehole in an approximately circular distribution. With eccentric charging, cracks preferentially initiate on the coupled side with higher density and exhibit symmetry only about the vertical axis.
(3) Multi-cartridge arrangements outperform single-cartridge layouts in rock fragmentation, and the optimal charge structure varies with the number of cartridges. Under the same total charge weight: two-cartridge arrangements achieve higher stress levels and damage ranges than single-cartridge layouts, with bottom eccentric charging yielding the best results. Three-cartridge arrangements further improve fragmentation uniformity, with concentric charging achieving the highest damage area ratio and more efficient energy utilization.
(4) Quantitative damage analysis confirms the distinct advantages of specific charge structures. The damage area ratio analysis reveals that: single-cartridge bottom eccentric charging improves outcomes by approximately 15 ~ 20% compared to concentric charging; two-cartridge bottom eccentric charging enhances results by about 25 ~ 30%; and three-cartridge concentric charging outperforms eccentric charging by roughly 20 ~ 25%. Therefore, in practical engineering, single- and two-cartridge setups are recommended to adopt bottom eccentric charging to enhance localized fragmentation, while three-cartridge arrangements should employ concentric charging to achieve uniform rock fragmentation.

## References

[1] Ocak I, Bilgin N. Comparative studies on the performance of a roadheader, impact hammer and drilling and blasting method in the excavation of metro station tunnels in Istanbul. Tunnelling and Underground Space Technology. 2010;25(2):181–7. doi: 10.1016/j.tust.2009.11.002

[2] Zhang Z, Zhou C, Remennikov A, Wu T, Lu S, Xia Y. Dynamic response and safety control of civil air defense tunnel under excavation blasting of subway tunnel. Tunnelling and Underground Space Technology. 2021;112:103879. doi: 10.1016/j.tust.2021.103879

[3] Ji L, Zhou C, Lu S, Jiang N, Li H. Modeling study of cumulative damage effects and safety criterion of surrounding rock under multiple full-face blasting of a large cross-section tunnel. International Journal of Rock Mechanics and Mining Sciences. 2021;147:104882. doi: 10.1016/j.ijrmms.2021.104882

[4] Xie LX, Lu WB, Zhang QB, Jiang QH, Wang GH, Zhao J. Damage evolution mechanisms of rock in deep tunnels induced by cut blasting. Tunnelling and Underground Space Technology. 2016;58:257–70. doi: 10.1016/j.tust.2016.06.004

[5] Verma HK, Samadhiya NK, Singh M, Goel RK, Singh PK. Blast induced rock mass damage around tunnels. Tunnelling and Underground Space Technology. 2018;71:149–58. doi: 10.1016/j.tust.2017.08.019

[6] Mandal SK, Singh MM, Dasgupta S. Theoretical Concept to Understand Plan and Design Smooth Blasting Pattern. Geotech Geol Eng. 2008;26(4):399–416. doi: 10.1007/s10706-008-9177-4

[7] Zou B, Xu Z, Wang J, Luo Z, Hu L. Numerical Investigation on Influential Factors for Quality of Smooth Blasting in Rock Tunnels. Advances in Civil Engineering. 2020;2020(1). doi: 10.1155/2020/9854313

[8] Zhang J. Stress-field research and application of eccentric decouple charge. Industrial Safety and Environmental Protection. 2001;27(8):20–3.

[9] Pu C. Test and research on eccentric decouple charge blasting. Chemical Minerals and Processing. 2007;36(4):30–43.

[10] Taylor LM, Chen E-P, Kuszmaul JS. Microcrack-induced damage accumulation in brittle rock under dynamic loading. Computer Methods in Applied Mechanics and Engineering. 1986;55(3):301–20. doi: 10.1016/0045-7825(86)90057-5

[11] Grady DE, Kipp ME. Continuum modelling of explosive fracture in oil shale. International Journal of Rock Mechanics and Mining Sciences & Geomechanics Abstracts. 1980;17(3):147–57. doi: 10.1016/0148-9062(80)91361-3

[12] Dehghan Banadaki MM, Mohanty B. Numerical simulation of stress wave induced fractures in rock. International Journal of Impact Engineering. 2012;40–41:16–25. doi: 10.1016/j.ijimpeng.2011.08.010

[13] Pan C, Li X, Li J, et al. Numerical investigation of blast-induced fractures in granite: Insights from a hybrid LS-DYNA and UDEC grain-based discrete element method. Geomechanics and Geophysics for Geo-Energy and Geo-Resources. 2021;7(2):1–18.

[14] Rong H, Li N, Cao C, Wang Y, Li J, Li M. Numerical simulation of blasting behavior of rock mass with cavity under high in-situ stress. Sci Rep. 2024;14(1):16046. doi: 10.1038/s41598-024-67088-5 38992235 PMC11239945

[15] Rong H, Li N, Cao C, Wang Y, Li J, Li M. Numerical simulation of rock blasting under different in-situ stresses and joint conditions. PLoS One. 2024;19(4):e0299258. doi: 10.1371/journal.pone.0299258 38648218 PMC11034639

[16] Li X, Liu K, Yang J. Study of the Rock Crack Propagation Induced by Blasting with a Decoupled Charge under High In Situ Stress. Advances in Civil Engineering. 2020;2020(1). doi: 10.1155/2020/9490807

[17] Yuan W, Wang W, Su X, Wen L, Chang J. Experimental and numerical study on the effect of water-decoupling charge structure on the attenuation of blasting stress. International Journal of Rock Mechanics and Mining Sciences. 2019;124:104133. doi: 10.1016/j.ijrmms.2019.104133

[18] Huo X, Qiu X, Shi X, Chen H, Zong C, Xie C. Attenuation Characteristics of Blasting Stress Under Decoupled Cylindrical Charge. Rock Mech Rock Eng. 2023;56(6):4185–209. doi: 10.1007/s00603-023-03286-3

[19] Chen M, Ye Z, Lu W, Wei D, Yan P. An improved method for calculating the peak explosion pressure on the borehole wall in decoupling charge blasting. International Journal of Impact Engineering. 2020;146:103695. doi: 10.1016/j.ijimpeng.2020.103695

[20] Wang Y. Study of the dynamic fracture effect using slotted cartridge decoupling charge blasting. International Journal of Rock Mechanics and Mining Sciences. 2017;96:34–46. doi: 10.1016/j.ijrmms.2017.04.015

[21] Wang Y, Wen Z, Liu G, Wang J, Bao Z, Lu K, et al. Explosion propagation and characteristics of rock damage in decoupled charge blasting based on computed tomography scanning. International Journal of Rock Mechanics and Mining Sciences. 2020;136:104540. doi: 10.1016/j.ijrmms.2020.104540

[22] Yang R, Ding C, Yang L, Lei Z, Zheng C. Study of decoupled charge blasting based on high-speed digital image correlation method. Tunnelling and Underground Space Technology. 2019;83:51–9. doi: 10.1016/j.tust.2018.09.031

[23] Wang Y, Fu D, Shen Y. Research on vibration response characteristics of circumferential water coupling blasting seismic exploration technology in loess layer areas. Journal of China Coal Society. 2025;50(S1):449–61.

[24] Fan Y, Wu F, Leng Z, et al. Peak elimination effect of radial uncoupled charge on explosion pressure and its influence on rock fracture range. Acta Armamentarii. 2024;45(01):131–43.

[25] Zhang X, Liu X, Wang X. Influence of blast hole spacing on penetrating crack propagation of eccentric uncoupled charge. Journal of Shandong University of Science and Technology (Natural Science). 2024;43(03):1–10.

[26] Jin Y, Yao Y, Liu W. Numerical simulation study of borehole wall pressure and rock dynamic response under eccentric uncoupled charge. Hazard Control in Tunnelling and Underground Engineering. 2025;7(03):83–92.

[27] Wang Y, Kong W, Wang D. Analysis of detonation wave effect in radial water coupling blasting. Journal of China Coal Society. 2024;49(S2):782–99.

[28] Wang P, Huang A, Zheng X, Zhou S. Numerical Study of Asymmetry in Blast Pressure Propagation and Rock Damage Under Eccentric Decoupled Charges. Symmetry. 2025;17(9):1583. doi: 10.3390/sym17091583

[29] Chi LY, Xu X, Zhang Z-X, Yang J. Strain Field Development, Fracturing, and Gas Ejection in Decoupled Charge Blasting Using Granite Cylinders. Rock Mech Rock Eng. 2024;57(11):10133–51. doi: 10.1007/s00603-024-04079-y

[30] Dai J. Rock dynamic characteristics and blasting theory. Metallurgical industry press; 2002.

[31] Zong Q. Calculation of radius of rupture zone of explosion stress wave in rock. Blasting. 1994;11(2):15–7.

[32] Dai J. Calculation of rock crushing circle and fracture circle in cylindrical charge blasting. Journal of Liaoning University of Engineering and Technology: Natural Science Edition. 2001;20(02):144–7.

[33] Bi C, Wang Z, Shi G. The application of initial volume fraction method in explosion simulation. Engineering Blasting. 2017;23(04):26–33.

[34] Shao P, Dong Z, Zhang Y. Review of rock blasting models. Rock and Soil Mechanics. 1999;03:91–6.

[35] Li X, Zhu Z, Wang M, Wan D, Zhou L, Liu R. Numerical study on the behavior of blasting in deep rock masses. Tunnelling and Underground Space Technology. 2021;113:103968. doi: 10.1016/j.tust.2021.103968

[36] Wang Z, Wang H, Wang J, Tian N. Finite element analyses of constitutive models performance in the simulation of blast-induced rock cracks. Computers and Geotechnics. 2021;135:104172. doi: 10.1016/j.compgeo.2021.104172

[37] Hallquist J. Keyword user’s manual. California: Livermore Software Technology, 2012.

[38] Banadaki MMD. Stress-wave induced fracture in rock due to explosive action. 2010.

[39] Yang R, Chen J, Xiao C. A method to calculate the dynamic compressive strength of rock with single specimen. Journal of Vibration and Shock. 2018;37(4):123–7.

[40] Li X, Li H, Liu K. Research on the dynamic properties and fracture characteristics of rocks subject to impact loading. Chinese Journal of Rock Mechanics and Engineering. 2017;36(10):2393–405.

[41] Zong Q, Cheng B, Wang H. Numerical simulation of pressure on borehole wall and damage effect with eccentric decouple charge. Blasting. 2019;36(1):76–83.

